# Supplementary material for: Enabling 3D CT-scanning of cultural heritage objects using only in-house 2D X-ray equipment in museums
Source: Nat Commun. 2024 May 14;15:3939. doi: 10.1038/s41467-024-48102-w (PMC11094032; doi:10.1038/s41467-024-48102-w)
Supplement: Supplementary file 1 — Supplementary Information [file 41467_2024_48102_MOESM1_ESM.pdf]

Supplementary Information

This document is the Supplementary Information to ‘Enabling 3D CT-scanning of cultural heritage objects using only in-house 2D X-ray equipment in museums’, Bossema et al. (Nature Communications, 2024). In this document we extend upon the methods and implementation. For full information please refer to the main text.

A Supplementary Methods

A.1 Model and optimisation

In this section we discuss the theory and model that underlies the implementation of our marker-based system parameter derivation. In less sophisticated X-ray imaging equipment, these parameters are often not available. By including small metal balls in the acquisition, we retrieve these parameters based on the radiographs. Each marker is visible as a circle on all (or most) projections, and we call its location the projected marker location (PML). For each marker we measure the PML on the X-ray images. Given a set of system parameters and marker positions, and for each marker at each rotation angle, we can draw a line from the source through the marker. The intersection of this line with the detector plane gives a predicted PML. The goal is to minimise the distance between the predicted PMLs and the measured PMLs. This will provide an estimated value for the system parameters, which we need for 3D reconstruction as described in section 4 in the main article. We aim to find parameters that can be used to produce a 3D reconstruction of the object. Our method does not aim to find the absolute physical distances. The only impact of this on the 3D reconstruction is the scale, which can if needed be obtained by including an object of known size in the scan or by measuring one distance on the object afterwards and scaling the reconstruction accordingly. In the next sections, we will first discuss the model used for the forward projection of marker positions and then the optimisation that is used for estimating the system parameters.

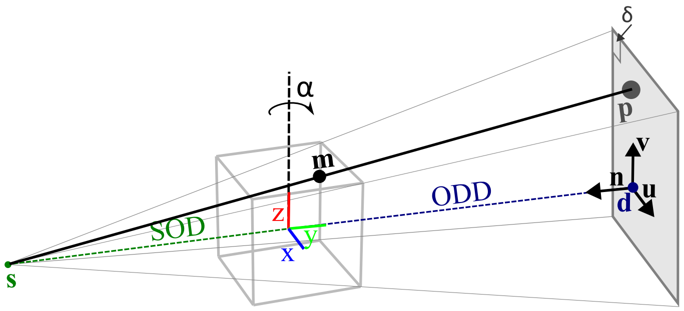

Supplementary figure 1. Schematic indicating the variables and notation used in the forward model.

A.1.1 The forward model

We assume that the setup consists of a cone-beam X-ray source, flat-panel detector and a rotation stage. A block of foam containing markers is mounted on the rotation stage next to the object. We assume that the components are stably mounted and do not drift during acquisition. For our calculations, we model the system as the source and detector rotating around the object, which is mathematically equivalent to a rotating object between a static source and detector.

We define a left handed coordinate system with the rotation axis being the z-axis. The y-axis is defined such that the source lies on it for the first projection. The first projection is defined as rotation angle 0°. The coordinate system and variables used to describe all the system components are shown in Supplementary figure 1.

A boldface small letter represents a vector with an x, y and z component, e.g. $\mathbf{s} = (s_x, s_y, s_z)$, which represents the location of the source. The detector plane is defined by a point $\mathbf{d}$, which is the center of the detector plane, the detector pixel size and the unit vectors $\mathbf{u}$ and $\mathbf{v}$ that span the detector plane. The normal vector to the detector plane is $\mathbf{n} = \mathbf{v} \times \mathbf{u}$. The out-of-plane rotations of the detector are given by angles θ (around axis $\mathbf{u}$) and ϕ (around axis $\mathbf{v}$). The parameter η defines the in-plane detector rotation (rotation around $\mathbf{n}$). With a subscript i, e.g. $\mathbf{s}_i$, we denote the vector that is obtained when rotating the original vector around the z-axis by angle $-\alpha_i$. The marker position of marker j is denoted by $\mathbf{m}_j = (m_{jx}, m_{jy}, m_{jz})$ for $j \in \{1, \ldots, N\}$.

A line from the source through marker j for projection i is given as $\mathbf{f}(t) = \mathbf{s}_i + t(\mathbf{m}_j - \mathbf{s}_i)$. The vector defining the projected 3D location in space of the PML corresponding to marker j on projection i, is given by the intersection of this line with the detector plane defined by $\mathbf{d}_i$ and $\mathbf{n}_i$: $\mathbf{g} = \mathbf{s}_i + \frac{\mathbf{n}_i \cdot (\mathbf{d}_i - \mathbf{s}_i)}{\mathbf{n}_i \cdot (\mathbf{m}_j - \mathbf{s}_i)}(\mathbf{m}_j - \mathbf{s}_i)$. This 3D location can be rewritten to the detector pixel on which the marker would fall. In other words, we obtain the PML $\mathbf{p}^{pred}_{i,j}(\Theta, \mathbf{m}_j) = (a, b)$ (row, column) such that $\mathbf{d}_i + a\mathbf{u}_i + b\mathbf{v}_i = \mathbf{g}$.

A.1.2 The cost function

The measured PML $\mathbf{p}^{meas}_{ij}$ of marker j on projection image i, is defined relative to $\mathbf{d}_i$ and is given by (c, d) (row, column).

The set of free parameters, which we for convenience denote by Θ, define the forward projections. The free parameters are given in Supplementary table 1 and the fixed parameters in Supplementary table 2. The source is fixed to the negative y-axis. We fix the detector pixel size, since this is often specified in the documentation of the manufacturer. We want to minimise the distance between the predicted PML $\mathbf{p}^{pred}_{ij}(\Theta, \mathbf{m}_j)$ and the measured PML $\mathbf{p}^{meas}_{ij}$ of marker j on projection image i. We therefore want to find the parameters Θ and $\mathbf{m}_j$, $j, \in \{1, \ldots, N\}$ that minimise the following:

$$\sum_i \sum_j |\mathbf{p}^{meas}_{ij} - \mathbf{p}^{pred}_{ij}(\Theta, \mathbf{m}_j)|^2. \tag{1}$$

Parameter	Notation
detector position	$\mathbf{d}$
detector tilt	θ, ϕ
detector in-plane rotation	η
projection angles	$\alpha_1, \ldots, \alpha_{n-1}$
marker positions	$\mathbf{m}_1, \ldots, \mathbf{m}_N$

Supplementary table 1. Notation of the free system parameters and marker positions $\mathbf{m}$ that are estimated in the optimisation scheme.

Parameter	Notation and fixed value
source position	$\mathbf{s}_0 = (0, SOD, 0)$
first projection angle	$\alpha_0 = 0$
detector pixel size	δ = detector pixel size

Supplementary table 2. Fixed system parameters. The source to object distance (SOD) and detector pixel size are user input.

A.2 Implementation

The proposed workflow consists of six steps (Fig. 2), the first two are the practical data acquisition phase which takes place in the X-ray suite:

1. Object and marker holder preparation;
2. Data acquisition;

After data acquisition the computational workflow consist of the following steps:

1. Marker detection and labelling;
2. System parameter derivation;
3. Pre-processing and Inpainting;
4. 3D reconstruction.

In the following sections we expand on each of these steps.

A.2.1 Data acquisition in the X-ray suite

Object and marker holder preparation
First, a marker holder is made by inserting a number of markers into two or more pieces of foam. The size and shape can be arbitrary, the holder can be adjusted to the size and shape of the object. They should be distributed vertically to limit the overlap of the projected markers on the radiographs.The object is mounted on the rotation stage, the marker holder is placed next to it. Using the live radiographic inspection, the positions of the markers are checked and adjusted if needed.

Although a full discussion of the requirements of the positions of the markers within the foam is beyond the scope of this article, here we give some general guidelines. For an accurate parameter estimation, the markers should be distributed within the foam in three dimensions so that they span the detector field of view where the object is located. If the markers are on one vertical line for example, the depth information is not captured in their positions as well as when they are distributed. This is due to the fact that the trajectory of the markers on the detector forms an ellipse and therefore movement of the PML (and the corresponding geometric information gain) is less on the sides of the ellipse than in the center. The number of markers needs to be sufficient for the system of equations to be resolved. In practice, it is advisable to take more markers where possible, since the contrast with the object may not always be sufficient to find all markers in each radiograph or they rotate out of the field of view for a

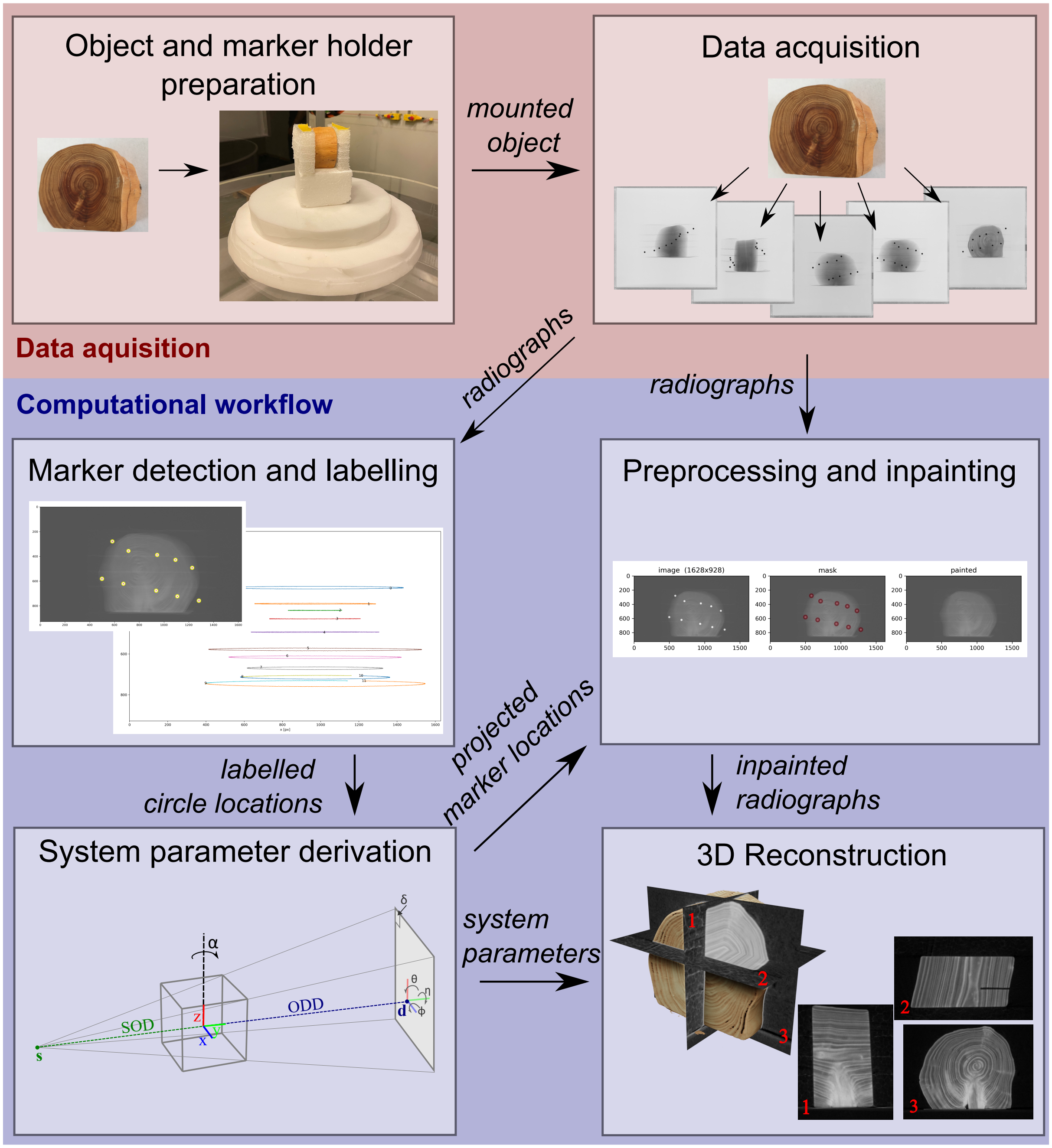

Supplementary figure 2. Steps in the post-scan marker-based parameter derivation method for 3D reconstruction.

few radiographs. The overlap of markers on the radiographs should be avoided, because this hampers the correct labelling of the markers. Therefore, we distribute the markers vertically to limit overlap on radiographs. Since the rotation angles are part of the parameter set, in each radiograph markers should be present. For the scans in the main text, we have used a minimum distance between markers of 1cm. We have moreover used the live radiographic inspection to ensure as few projections as possible had overlapping markers. This facilitates the labelling and tracking of the markers. For the wooden block 10 markers were used and for the case study 17. The following steps can be used to setup the markerholder:

1. Place the markers in two or more pieces of foam, keeping them at least 1cm apart and avoiding placing them on a straight line. The number of markers will depend on the size of the object and the magnification, since the main goal is to have no overlapping markers on the radiographs. See Supplementary figure 3a.
2. Place the pieces of foam on the rotation stage, next to the mounted object. If convenient, placing two at 90°gives a good spread of the markers on the detector view. See Supplementary figure 3b,c.
3. Turn on live radiographic inspection of the object and markers and determine the maximum magnification that ensures the object stays in the field of view during rotation. See Supplementary figure 3d.
4. Make sure that during rotation there is no radiograph on which no markers are visible. Markers can rotate out of the field of view, but in every radiograph markers need to be present.
5. If there are radiographs in which the markers overlap, try to increase the vertical space between markers until they do not overlap.

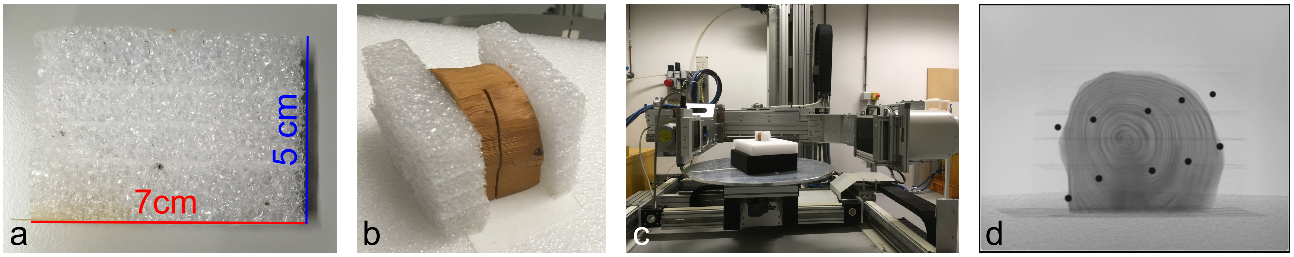

Supplementary figure 3. Preparing the markerholder. a) Including markers in foam. b) Placing foam next to the wooden block. c) Placing the wooden block and markerholder on the rotation stage at the Rijksmuseum. d) Resulting radiograph in the Rijksmuseum facility.

Data acquisition

Radiographs are collected over one or more revolutions of the rotation stage. A flatfield, an image with the source turned on without an object in view, and darkfield, an image with the source off, are collected for the pre-processing step[1].

A.2.2 Computational workflow

Marker detection and labelling

The computational workflow starts with measuring the PML on the projections, by first using the Canny Edge detector[2] and consequently an implementation of the Hough Transform to identify circles from the Scikit toolbox[3]. Making use of the Trackpy toolbox[4], we identify the same projected marker from projection to projection, forming the ellipse shaped trajectory fol-

lowed by the projected marker (step 2 in Fig. 2). Both locating and labelling may require user input, as the locating of projected marker on the image depends on the total brightness and contrast and wrongly labelled projected markers can make parameter estimation less reliable. During the locating step some dense features in the object may be identified as projected markers that do not correspond to markers. When the markers overlap with the object or rotate out of the field of view, the corresponding projected markers may not be found in a number of projections. This causes errors in the labelling step. These errors are reduced by filtering out short trajectories, determining a maximum step size for the projected marker from projection to projection and a memory parameter that determines for how many projections a marker can be missing to still belong to the same label. These parameters can be influenced by the user based on visual inspection of the resulting trajectories. It is preferable to have a trajectory cut into multiple labels over mislabelling (e.g. crossover of labels between two markers). The number of found labels is N^*. Note that this can be a higher number than the actual number of markers N, due to features in the object being identified as projected markers or partially labelled trajectories, or a lower number due to non-identified projected markers in the locating step. During the optimisation in the next step this is taken into account.

System parameter estimation

The System parameter estimation algorithm is given in Algorithm 1. It consist of two optimisation steps with a marker merging step in between.

The required user input is the detector pixel size (δ) and an estimation of the source to object distance (SOD). This source position is used to fix the location of the source at distance SOD from the rotation axis on the negative y-axis (see Section A.1.1). The resulting reconstruction will therefore be scaled relative to this given SOD.

To provide an educated initial guess on the system parameters, further input is requested from the user: estimated values for the ODD, the number of revolutions (n_{rounds}) of the rotation stage and the approximate average distance r of the markers to the rotation center. These inputs are used to create the following initialisation: i) The detector is placed on the positive y-axis at distance ODD from the origin, ii) the detector tilts and skew are 0, iii) projection angles are equidistant over $2\pi n_{rounds}$ (radians) and iv) the initial marker locations are placed randomly within a ball with radius r, their labels based on their vertical location. This initial guess and the residual function described by equation 1 are input for the *scipy.optimize* package's *least_squares* function[5]. Derivatives are calculated using an automated derivative package *autograd*[6], that is a wrapper for *numpy*[7].

For each iteration a reduced number of projected markers per projection image are used, to make the method robust against mislabelled markers. Projected markers are selected that have the lowest distance of the predicted PML to the measured PML. In other words, we use labels $h_i(\Theta, N_k)$, which are the N_k labels j with smallest $|\mathbf{p}_{ij}^{meas} - \mathbf{p}_{ij}^{pred}(\Theta, \mathbf{m}_j)|$. The number of projected markers that are used in the first and second optimisation step are given by N_1 and N_2 and are input by the user. The least squares optimisation terminates when the step size or cost function improvement are below a given threshold of 10^{-6}. The user can give an upper limit n_{iter1} and n_{iter2} for the number of iterations of the least squares solver in the first and second optimisation step respectively. In the marker merging step a minimum distance between marker positions is used to decide whether or not to merge two labels. This distance can be chosen by the user.

Algorithm 1 System parameter estimation.

1: **Initial Guess.** The initial guess is defined by a standard circular scan with equidistant angles and the user input values ODD, n_{rounds} and r.

2: **First optimisation.** Run least squares optimisation starting from the initial guess until thresholds are reached or until the number of iterations exceeds n_{iter1}, to find the parameters (Θ^* and $\mathbf{m_1^*} \ldots, \mathbf{m_N^*}$) that minimise the following value

$$\sum_i \sum_{j \in h_i(\Theta^*, N_1)} |\mathbf{p}_{ij}^{meas} - \mathbf{p}_{ij}^{pred}(\Theta^*, \mathbf{m}_j^*)|^2.$$

3: **Merge markers.** *If* the distance between the positions of two markers $|\mathbf{m}_j - \mathbf{m}_k|$ is smaller than a given threshold *and* do not overlap for more than a given number of frames, the labels j, k refer to the same projected marker and their trajectories are merged, or in other words k is relabelled j and removed. N^{**} denotes the number of labels after this merging step.

4: **Updated initial guess** Use Θ^* and $\mathbf{m}_j^*$, $j \in \{1, \ldots, N^{**}\}$ as updated initial guess for the second optimisation step.

5: **Second optimisation.** Run least squares optimisation starting from the initial guess until thresholds are reached or until the number of iterations exceeds n_{iter2}, to find the parameters (Θ^{**} and $\mathbf{m_1^{**}} \ldots, \mathbf{m_N^{**}}$) that minimise the following value

$$\sum_i \sum_{j \in h_i(\Theta^{**}, N_2)} |\mathbf{p}_{ij}^{meas} - \mathbf{p}_{ij}^{pred}(\Theta^{**}, \mathbf{m}_j^{**})|^2.$$

6: **Calibrated parameters.** Return Θ^{**}.

Pre-processing and inpainting

The recorded data is first flat- and darkfield corrected. Using the system parameters and marker positions found in the previous step, a forward projection is performed to obtain the predicted PML locations on all the projections. These are used to algorithmically remove the projections of the markers on the radiographs by the inpainting function of the scikit-image package[8], because high density material in a CT acquisition can cause image artefacts in the reconstruction[9]. It is possible to perform both a reconstruction with the original radiographs and the inpainted radiographs. Therefore the user can choose which reconstruction serves them best, since the effect of inpainting can differ per object and placement of the markers.

3D reconstruction

The inpainted projections, together with the system parameters resulting from the optimisation step are used to obtaining a 3D reconstruction. Because of the fixed source position, the solution that is obtained, is a scaled reconstruction. The estimated system parameters are transformed into a geometry description that is then used within the SIRT algorithm provided by the FleX-box toolbox[10] to make a 3D reconstruction of the object.

B Supplementary Figures and Tables

B.1 Parameters

In Supplementary table 3 the computed system parameters of the scans of the wooden block are given. In Supplementary figure 4 the found angles for each system are given. We see that the British Museum setup provides equidistant angles and the other two systems have less regular angular intervals.

System parameters	**BM system**	**BM markers**	**GM markers**	**RM markers**	FleX-ray
source location (mm)	(0,881,0)	(0,881,0)	(0, 881, 0)	(0,500,0)	(0,658.02,0)
detector location (mm)	(-31.46, 1362.0, 0)	(-31.32, 1471.44, -3.84)	(-32.97, 1351.04, -6.71)	(-2.63, 583.97, 2.78)	(0,430.98,0)
detector tilts (radians)	0, 0	0.027, 0.019	-0.004, 0.015	0.021, 0.001	0.0, 0.0
detector in-plane rotation	0.0	0.0	0.0	-0.002	-0.004

Supplementary table 3. System parameters as reported by the system feedback (BM system) or the marker-based parameter retrieval (BM markers, GM markers, RM markers) for the scans of the wooden block (main article section 2.2). The source location is an estimate given by the user.

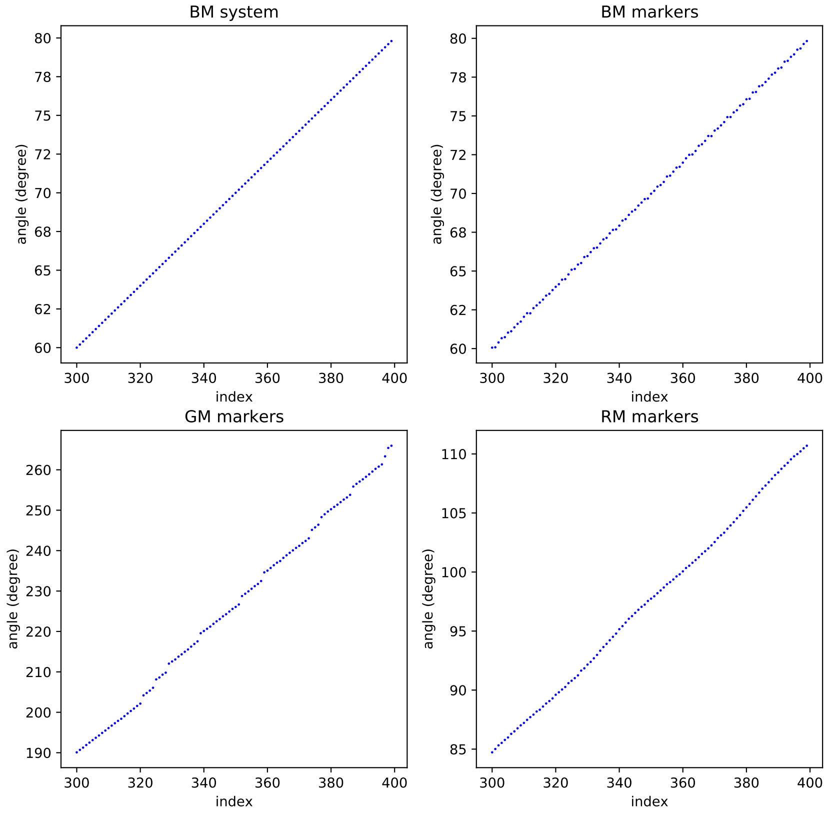

Supplementary figure 4. Subset of the acquisition angles (index vs. angle (degrees)) of the test scans of the wooden block (main article section 2.2), given by the British Museum system (BM system) and calculated by the marker-based parameter derivation at the British Museum (BM markers), The J. Paul Getty Museum (GM markers) and the Rijksmuseum (RM markers).

To investigate the accuracy and precision of the found marker positions, we performed simulation experiments. We simulated projected marker locations (PMLs) by forward projecting 3D marker locations. To simulate an incorrectly found center of the PML, we added gaussian noise with mean 0 and standard deviation from 0 up to 5 pixels to the PML. Next, we ran our parameter retrieval optimisation to obtain estimated 3D marker locations. Since our method does not assume the projection angles and other system parameters are known, the found 3D marker locations may have a slightly different orientation, vertical position, and scaling compared to the original marker positions. These variations do not affect the reconstruction quality, so to be able to measure the quality of the found marker locations, we compensate for them before comparing the found marker locations with the original marker locations used for the forward projection. For each choice of standard deviation, we ran this simulation with ten different random seeds, which influences the noise added to the PMLs, to obtain Supplementary figure 5 showing standard deviation (in pixels) versus the average error in the calculated marker positions (in mm). The average error is lower than the voxel size (0.13mm) except for a few outliers. In the practical datasets included in the manuscript, we found that the PML identification can be trusted to locate the centers within this error range.

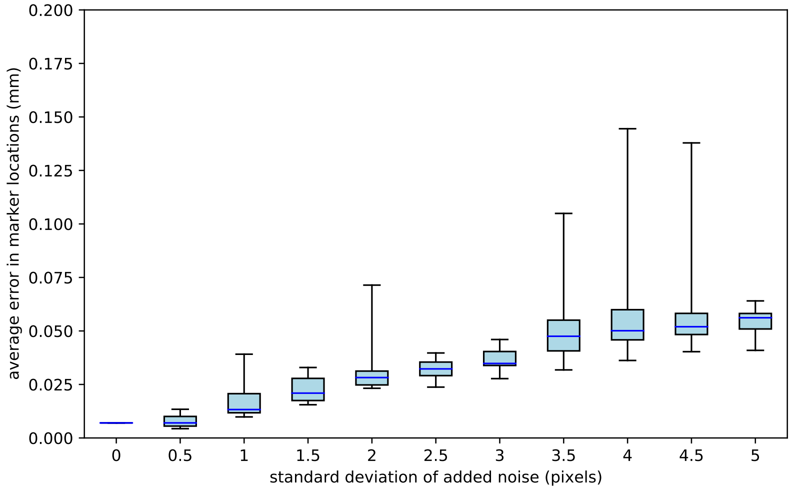

Supplementary figure 5. Results of the simulation experiment. Boxplot showing average errors in the calculated marker positions (y-axis) when adding gaussian noise to the PML (standard deviation of noise on the x-axis). The boxplot shows the median (blue line), interquartile range (lightblue box), which shows where 50% of the data points around the median fall, and minima and maxima of the data (black horizontal bars).

B.2 Comparison of the marker based approach and FDK

Here we show the type of image artefacts that can be encountered when an imperfect calibration is used for a CT reconstruction. In Supplementary figure 6 a slice from a reconstruction of the wooden block dataset recorded at the J. Paul Getty museum is shown using the approach outlined in the main text and using a straightforward FDK approach with the angles estimated to be equidistant. The irregularity of the angular interval produces wrongly back-projected radiographs in the second reconstruction, showing the need for estimating the individual angular intervals. In Figure 7 we show the effect of errors in the rotation speed. Here, these effects are shown on a dataset of the wooden block recorded at a micro-CT facility, the FleX-ray laboratory, located at the Center for Mathematics and Computer Science in Amsterdam. For Figure 7 we removed angles at the end of the full rotation to simulate a slower rotation speed.

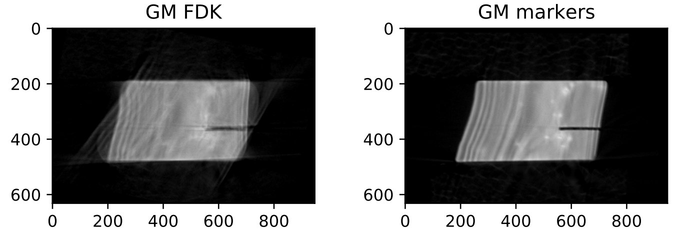

Supplementary figure 6. Comparing the marker-based parameter retrieval method with a straightforward FDK reconstruction. A slice from the reconstruction of the dataset of the wooden block at the J. Paul Getty Museum with a) marker-based parameter retrieval and b) FDK with the angles estimated to be equidistant.

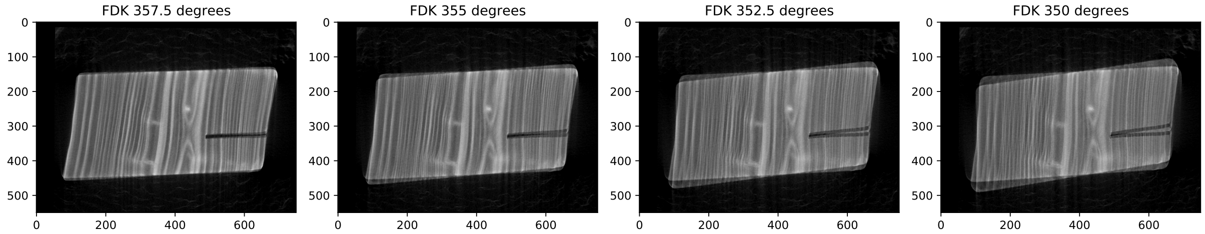

Supplementary figure 7. Effect of lower rotation speed on FDK reconstruction. Here we show an FDK reconstruction where the rotation stage does not fully rotate to 360 degrees during one acquisition, reaching from left to right only 357.5, 355,352.5 and 350 degrees, respectively, while the reconstruction incorrectly assumes that the rotation was over the full 360 degrees. The slower the rotation stage the larger the effect on the FDK reconstruction.

B.3 Inpainting

In Supplementary figure 8 we show a radiograph from the dataset of the wooden block, recorded at the FleX-ray laboratory, the mask used for inpainting and the inpainted radiograph. In Supplementary figure 9 a slice from a reconstruction of the wooden block dataset recorded at the FleX-ray laboratory is shown using the original radiographs and the inpainted radiographs. The effect of the inpainting on the reconstruction of the wooden block is visible in a blurring on the left side of the wooden block. The effect of the inpainting is dependent on the magnification. Since this is a high resolution scan, the magnification is large and therefore the marker shades a larger portion of the object than when the magnification is smaller. In Supplementary figure 10 we show the effect of the inpainting on the dataset recorded at the J. Paul Getty museum. Here the effect is small. Thus, the effect of inpainting depends on the settings. Whether to use the original or inpainted radiographs can be decided by the user upon inspection of the reconstructions.

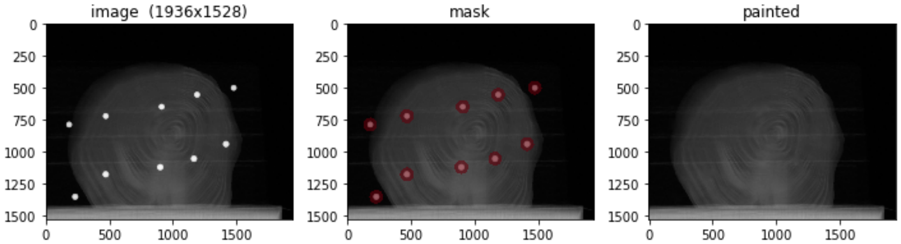

Supplementary figure 8. Inpainting of the radiographs. A radiograph of the wooden block at the FleX-ray laboratory: original radiograph (left) and an overlay of the mask used for inpainting (in red) on the radiograph (middle) and resulting inpainted radiograph (right).

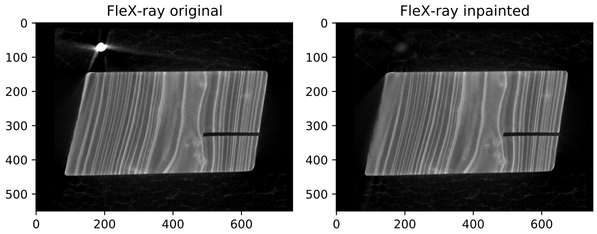

Supplementary figure 9. Comparing reconstructions with and without inpainting. A slice from the reconstruction of the dataset of the wooden block at the FleX-ray laboratory with original radiographs (left) and inpainted radiographs (right).

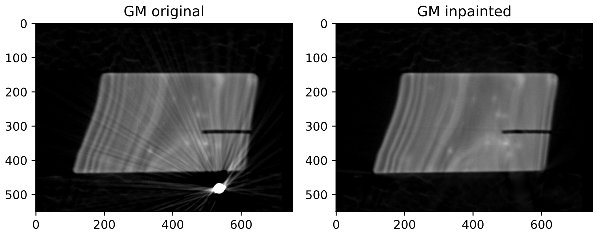

Supplementary figure 10. Comparing reconstructions with and without inpainting. A slice from the reconstruction of the dataset of the wooden block at the J. Paul Getty museum, using the marker based approach with a) original radiographs and b) inpainted radiographs.

Supplementary References

[1] Kharfi, F. Mathematics and Physics of Computed Tomography (CT): Demonstrations and Practical Examples in *Imaging and Radioanalytical Techniques in Interdisciplinary Research - Fundamentals and Cutting Edge Applications* (IntechOpen, 2013).

[2] *Scikit-image - Canny Edge detector* `https://scikit-image.org/docs/dev/auto_examples/edges/plot_canny.html` (Accessed: 14 Aug 2023).

[3] *Scikit-image - Hough_circle* `https://scikit-image.org/docs/stable/api/skimage.transform.html#skimage.transform.hough_circle` (Accessed: 14 Aug 2023).

[4] *Trackpy* `http://soft-matter.github.io/trackpy/v0.3.0/index.html` (Accessed: 14 Aug 2023).

[5] *Scipy Optimize - Least_squares* `https://docs.scipy.org/doc/scipy/reference/generated/scipy.optimize.least_squares.html` (Accessed: 14 Aug 2023).

[6] *Autograd* `https://github.com/HIPS/autograd` (Accessed: 14 Aug 2023).

[7] *Numpy* `https://numpy.org/` (Accessed: 14 Aug 2023).

[8] *Scikit-image - Inpainting* `https://scikit-image.org/docs/stable/auto_examples/filters/plot_inpaint.html` (Accessed: 14 Aug 2023).

[9] Schulze, R., Heil, U., Gross, D., Bruellmann, D., Dranischnikow, E., Schwanecke, U. & Schoemer, E. Artefacts in CBCT: a review. *Dentomaxillofacial Radiology* **40**, 265–273. (2011).

[10] Kostenko, A., Palenstijn, W.J., Coban, S.B., Hendriksen, A., van Liere, R. & Batenburg, K.J. Prototyping X-ray tomographic reconstruction pipelines with FleXbox. *SoftwareX* **11**, 100364. (2020).
